# Presynaptic Localization of Smn and hnRNP R in Axon Terminals of Embryonic and Postnatal Mouse Motoneurons

**DOI:** 10.1371/journal.pone.0110846

**Published:** 2014-10-22

**Authors:** Benjamin Dombert, Rajeeve Sivadasan, Christian M. Simon, Sibylle Jablonka, Michael Sendtner

**Affiliations:** Institute for Clinical Neurobiology, University Hospital Wuerzburg, Wuerzburg, Germany; Virginia Tech Carilion Research Institute, United States of America

## Abstract

Spinal muscular atrophy (SMA) is caused by deficiency of the ubiquitously expressed survival motoneuron (SMN) protein. SMN is crucial component of a complex for the assembly of spliceosomal small nuclear ribonucleoprotein (snRNP) particles. Other cellular functions of SMN are less characterized so far. SMA predominantly affects lower motoneurons, but the cellular basis for this relative specificity is still unknown. In contrast to nonneuronal cells where the protein is mainly localized in perinuclear regions and the nucleus, Smn is also present in dendrites, axons and axonal growth cones of isolated motoneurons *in* *vitro*. However, this distribution has not been shown *in* *vivo* and it is not clear whether Smn and hnRNP R are also present in presynaptic axon terminals of motoneurons in postnatal mice. Smn also associates with components not included in the classical SMN complex like RNA-binding proteins FUS, TDP43, HuD and hnRNP R which are involved in RNA processing, subcellular localization and translation. We show here that Smn and hnRNP R are present in presynaptic compartments at neuromuscular endplates of embryonic and postnatal mice. Smn and hnRNP R are localized in close proximity to each other in axons and axon terminals both *in* *vitro* and *in* *vivo*. We also provide new evidence for a direct interaction of Smn and hnRNP R *in* *vitro* and *in* *vivo*, particularly in the cytosol of motoneurons. These data point to functions of SMN beyond snRNP assembly which could be crucial for recruitment and transport of RNA particles into axons and axon terminals, a mechanism which may contribute to SMA pathogenesis.

## Introduction

Proximal spinal muscular atrophy (SMA), the most common form of motoneuron disease in children and young adult, is caused by deficiency or loss of function of SMN [1]. The SMN protein is widely expressed and plays a central role in the formation of spliceosomal uridine-rich small nuclear ribonucleoprotein (U snRNP) complexes [2]. Recent studies with fly models have provided evidence that defects of pre-RNA splicing in sensory neurons contribute to the pathogenesis of SMA [3]. In SMA patients, motoneurons are primarily affected. Other organs and even most other types of neurons in the central and peripheral nervous system are spared or much less affected, thus raising the question about the molecular mechanisms underlying the high vulnerability of motoneurons. Degeneration and dysfunction of axon terminals at neuromuscular endplates is a prominent hallmark in SMA [4]–[8]. Weakness of the proximal musculature is an early feature in SMA patients and this correlates with defects in neurotransmission at neuromuscular junctions (NMJ) [9], [10]. Similar observations have been made in mouse models of SMA [11]–[14] and in isolated SMA type I motoneurons, which developed defects in presynaptic differentiation and axonal excitability [15].

In most cell types, the SMN protein is found both in the perinuclear cytoplasm, where spliceosomal snRNP complexes are assembled, and in specific structures within the nucleus called Gemini of coiled bodies (Gems) [16], where such RNP particles are regenerated and processed [17]. In cultured motoneurons Smn is also found in cytoplasmic granules within neuronal processes and in axon terminals [18]–[20]. For its function in the assembly of spliceosomal U snRNP particles Smn associates with Gemin 1 to 7 and Unrip [21]–[23]. This complex assembles Sm core proteins and small nuclear RNAs into snRNPs [24], [25]. SnRNP particles are then transported back into the nucleus [26]. This function of Smn appears crucial for all cell types. Full knockout of the murine *Smn* gene results in early lethality [27], which is compatible with disruption of such an essential cellular function of Smn. The presence of a second *SMN* gene (*SMN2*) in humans gives rise to low amounts of functional SMN protein. This specific condition appears responsible for the observation that most organs are unaffected, whereas motoneurons become dysfunctional and degenerate [28]. Recent *in* *vitro* studies have detected Smn in association with components of the classical SMN complex, such as Gemin 2 and 3, in axons of cultured motoneurons and other types of neurons [15], [20]. However, Smn also associates with several other proteins which are not part of the SMN complex like HuD or hnRNP R [18], [19], [25], [29], the fragile X mental retardation protein (FMRP) [30], the ALS-related proteins FUS and TDP43 [31]–[34], and several other members of the heterogeneous nuclear ribonucleoprotein family [35]. These complexes bind mature mRNA species in motoneurons [20], [36], including ß-actin mRNA [19]. The interaction of Smn and hnRNP R appears particularly interesting since knockdown of hnRNP R in zebra fish or in isolated motoneurons [29] causes similar defects in motor axon growth as the depletion of Smn [37], indicating that the interaction of these two proteins is relevant in the context of axonal defects and dysfunction of axon terminals in SMA.

However, these studies did not provide an answer on whether Smn is also present in axons and axon terminals of developing and postnatal motoneurons *in* *vivo*, and whether the association with hnRNP R is direct and developmentally regulated. In order to address these questions, we studied the subcellular distribution and interaction of Smn and hnRNP R in motoneurons both *in* *vitro* and *in* *vivo*. We show here that Smn and hnRNP R interact directly with each other in the cytosol of motoneurons. Furthermore, we provide evidence that both proteins are present in axons and axon terminals of mouse motoneurons *in* *vitro* and *in* *vivo*, supporting the hypothesis that SMN is involved in the axonal translocation of hnRNP R and hnRNP R-bound protein/RNA particles, both during embryonic development and after birth.

## Results

### Localization of Smn and hnRNP R in isolated embryonic mouse motoneurons *in* *vitro*

**Figure 1:**
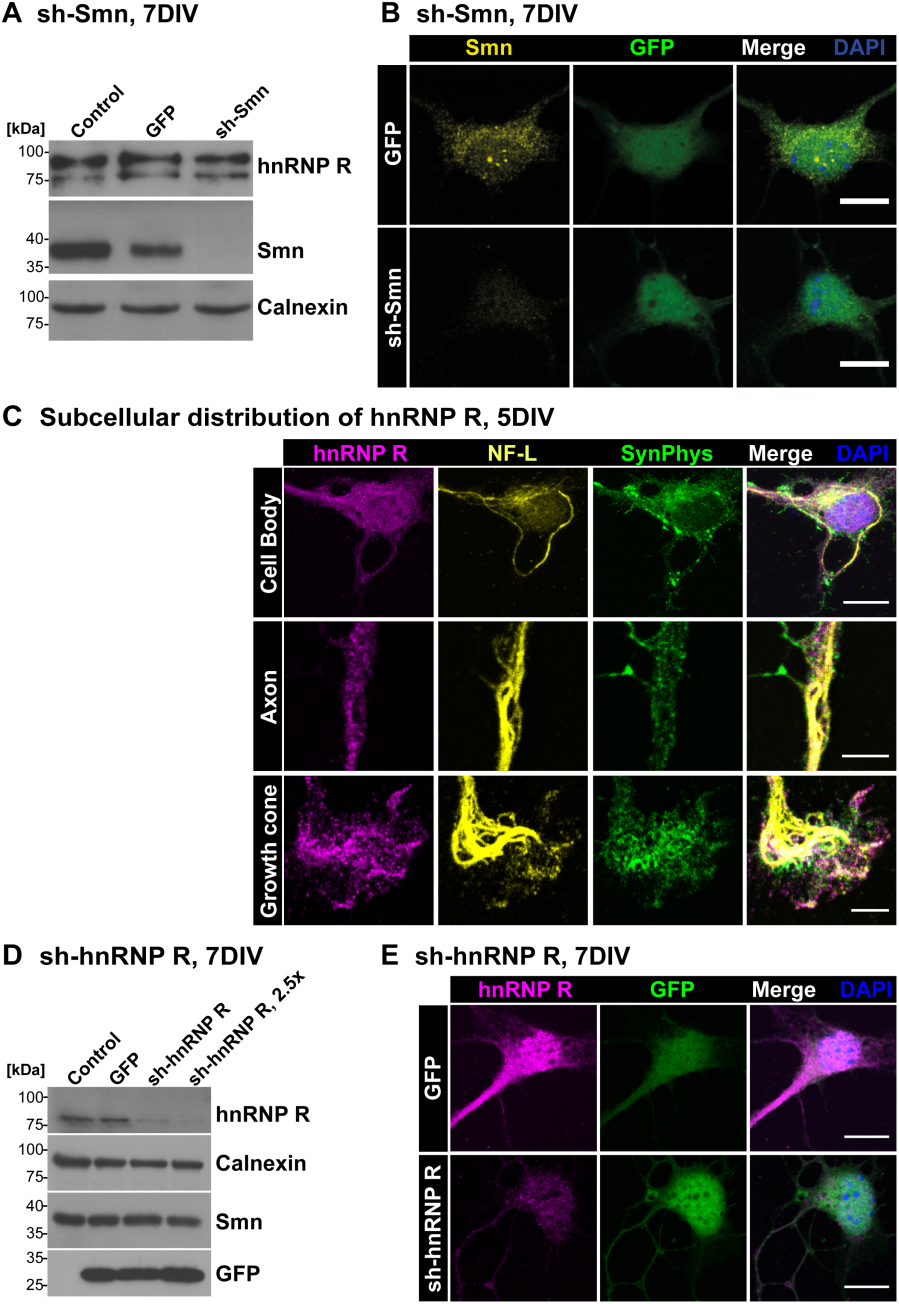
Subcellular distribution of Smn and hnRNP R in isolated embryonic motoneurons. (A) Motoneurons showed reduced Smn protein levels upon lentiviral knockdown of Smn. Uninfected or GFP-infected mouse embryonic motoneurons were used as controls. Levels of calnexin and hnRNP R were not affected. For this experiment a C-terminal antibody directed against hnRNP R was used as reported recently [29]. This antibody recognizes distinct hnRNP R isoforms. (B) Representative images of motoneurons cultured for 7DIV and labeled against Smn (scale bar: 10 µm). GFP-transfected controls revealed immunoreactive signals for Smn in the cytosol, in neuronal processes and in Gem-like nuclear structures. Upon lentiviral Smn knockdown both cytosolic Smn immunoreactivity (Uninfected set as ‘1’, n = 4, N = 51; GFP 1.02±0.04, n = 4, N = 60; sh-Smn 0.34±0.02, n = 4, N = 74; P<0.001, t = 19.19) and number of Gems per nucleus (Uninfected 1.03±0.18, n = 4, N = 51; GFP 0.97±0.15, n = 4, N = 60; sh-Smn 0.08±0.02, n = 4, N = 74; P<0.01, t = 4.929) were significantly reduced in comparison to uninfected cells. (C) Subcellular distribution of hnRNP R in soma, axon and growth cone of primary motoneurons cultured for 5DIV and costained against synaptophysin (SynPhys) and neurofilament (NF-L) (scale bar: 10 µm (upper row), 5 µm). (D) Lentiviral knockdown of hnRNP R led to a dose-dependent reduction of hnRNP R levels. Calnexin and Smn protein were not altered significantly. (E) HnRNP R knockdown was also detected by immunofluorescence validating the used antiserum peptide ICN 1-18 (GFP 1.00±0.04, n = 8, N = 100; sh-hnRNP R 0.48±0.04, n = 6, N = 63; P<0.0001, t = 8.719, DF = 12) (scale bar: 10 µm).

The assembly of spliceosomal U snRNPs (reviewed in [24], [38]) takes place in the cytoplasm surrounding the nucleus. This is the site where Smn normally is localized (reviewed in [39]) both in neuronal and nonneuronal cells. Smn is also found in nuclear structures called Gemini of coiled bodies (Gems) where spliceosomal U snRNPs are regenerated [17]. Furthermore, Smn is located in axons and axon terminals of isolated motoneurons [15], [20]. To confirm this subcellular distribution and to validate the antibodies used for Smn detection in this study, Smn immunoreactivity was investigated in primary motoneurons with and without lentiviral sh-mediated Smn knockdown. Western Blot analysis verified the specificity of the applied Smn antibodies showing a robust Smn depletion after shRNA-mediated knockdown (Fig. 1A). HnRNP R protein levels were not altered when Smn was deficient (Fig. 1A). Using the same antibody for immunofluorescent labeling of these motoneurons, Smn was found in nuclear Gem-like structures and in the cytosol (Fig. 1B). Motoneurons treated with sh-Smn revealed a significant reduction of mean Smn signal intensity of 66% (P<0.001, n = 4, N = 74) in the cytosol. Furthermore, the number of Smn-positive Gems per motoneuron cell body was reduced by 92% (0.08±0.02, n = 4, N = 74, P<0.01) in comparison to uninfected motoneurons (1.03±0.18, n = 4, N = 51). We did not detect any differences between uninfected and GFP-infected control cells (n = 4, N = 60) with respect to cytosolic Smn immunoreactivity (1.02±0.04) and number of Gems (0.97±0.15).

We then studied the localization of hnRNP R in isolated embryonic motoneurons. HnRNP R has multiple functions in transcription regulation and RNA processing (reviewed in [40], [41]). It interacts with Smn and shows high homology with hnRNP Q [18], [19], [42], [43]. HnRNP R depletion results in defective axon extension in primary mouse motoneurons and zebra fish [29] in a similar manner as Smn depletion [37], indicating that endogenous hnRNP Q cannot compensate for this function. Only the N-terminus of hnRNP R is distinct from hnRNP Q, and antibodies against this domain were used to distinguish both proteins [18] (Supplementary information, Fig. S1A). HnRNP R contains three consensus RNA-binding domains (RRM1-3) and an RGG-rich domain, which is typical for many proteins involved in RNA processing and transport (Fig. S1A). The antiserum directed against amino acid 1-18 of hnRNP R and termed herein ICN 1-18 (Fig. S1A) stained hnRNP R both in the nucleus and cytosol of these motoneurons (Fig. 1C). Relatively high levels of the protein were present in the nucleus when compared with Smn (Fig. 1B, C). Confocal microscopy of axons and growth cones revealed spot-like hnRNP R-immunoreactive structures (Fig. 1C). Antibodies against neurofilament light chain (NF-L) and synaptophysin (SynPhys) were used to visualize soma, axons and axon terminals, respectively. Western Blot analysis with the ICN 1-18 antiserum confirmed the lentiviral shRNA-mediated depletion of hnRNP R in a dose-dependent manner (Fig. 1D, S1B).

Immunofluorescence analysis after hnRNP R knockdown revealed also a significant decrease of hnRNP R signal in motoneuron cell bodies of 52% (P<0.0001, n = 6, N = 63) (Fig. 1E). To further characterize and verify the observed hnRNP R immunofluorescence we tested an additional antibody against the N-terminus of hnRNP R. This antibody revealed similar results with respect to distribution, localization and knockdown susceptibility (Fig. S1C). Western Blot analysis showed no significant reduction of Smn expression after hnRNP R depletion (Fig. 1D). The number of nuclear Smn-positive Gems and levels of cytosolic Smn immunoreactivity were also comparable between GFP-infected control and sh-hnRNP R-treated cells (Fig. S1C), as revealed by immunocytochemical analysis.

**Figure 2:**
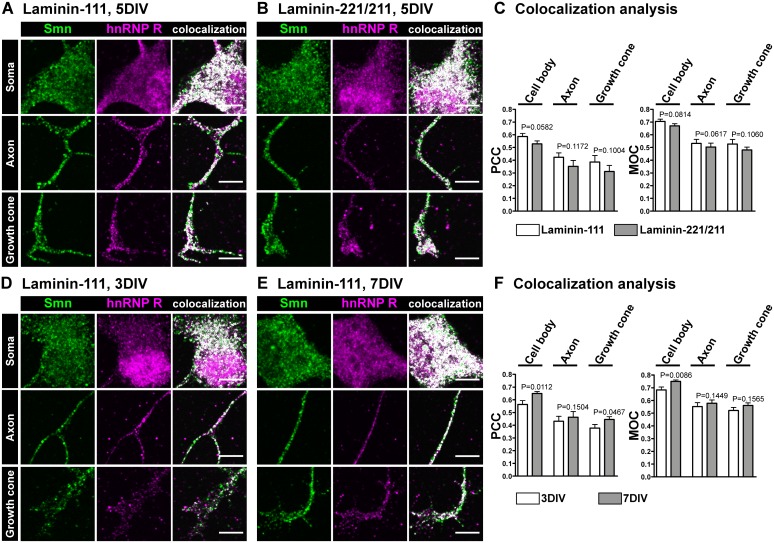
Colocalization of Smn and hnRNP R proteins in embryonic motoneurons. Representative images of cell bodies, axons and growth cones of primary embryonic motoneurons cultured on laminin-111 (A) and laminin-221/211 (B) for 5DIV and stained against Smn and hnRNP R (scale bar: 5 µm). Superimposed colocalizing points are highlighted in white. (C) No differences were observed with respect to colocalization and subcellular distribution of hnRNP R between these two investigated laminin isoforms. Representative images of cell bodies, axons and growth cones of motoneurons cultured on laminin-111 for either 3DIV (D) or 7DIV (E) and labeled against Smn and hnRNP R (scale bar: 5 µm). Both the degree of overlap between Smn and hnRNP R and the subcellular distribution of hnRNP R were regulated over time. The relative ratio of cytosolic versus nuclear hnRNP R immunoreactivity was significantly enhanced by 63% (P = 0.0173, t = 3.914, DF = 4) in motoneuron cell bodies cultured for 7DIV (1.63±0.16, n = 5, N = 46) in comparison to 3DIV (set as ‘1’; n = 5, N = 37). (F) After 7DIV (PCC 0.65±0.02, MOC 0.75±0.01, n = 5, N = 45) colocalization of Smn and hnRNP R in motoneuron cell bodies was higher (PCC P = 0.0112, t = 4.453, DF = 4; MOC P = 0.0086, t = 4.807, DF = 4) than after 3DIV (PCC 0.56±0.03, MOC 0.68±0.02, n = 5, N = 36). In axons the degree of overlap and correlation did not change (PCC P = 0.1504, t = 1.776, DF = 4; MOC P = 0.1449, t = 1.808, DF = 4) over time (3DIV PCC 0.43±0.04, MOC 0.55±0.03, n = 5, N = 36; 7DIV PCC 0.46±0.04, MOC 0.58±0.03, n = 5, N = 46), whereas in axonal growth cones a significant modification of the correlation (PCC P = 0.0467, t = 2.844, DF = 4; MOC P = 0.1565, t = 1.742, DF = 4) of both proteins was detected (3DIV PCC 0.38±0.03, MOC 0.52±0.02, n = 5, N = 37; 7DIV PCC 0.45±0.02, MOC 0.56±0.02, n = 5, N = 34).

Previous studies reported that Smn and hnRNP R can be coprecipitated from neuronal extracts [18], [19], [29]. To further corroborate and characterize this interaction we investigated potential colocalization and correlation of Smn and hnRNP R in cell body, axon and axonal growth cone of isolated embryonic mouse motoneurons by determining both the Pearson’s correlation coefficient (PCC) and the Manders Overlap Coefficient (MOC) (reviewed in [44]) (Fig. 2). In order to test whether signals for maturation of presynaptic terminals influence distribution and interaction of Smn and hnRNP R motoneurons were cultured either on laminin-111 (Fig. 2A) or synapse-specific laminin-221/211 (Fig. 2B) for 5DIV. Highest degrees of Smn/hnRNP R codistribution were found in the cell body, particularly in the perinuclear region, on laminin-111 (PCC 0.59±0.02; MOC 0.70±0.02; n = 6, N = 54) (Fig. 2A, C). In axons (PCC 0.42±0.03; MOC 0.53±0.03; n = 6, N = 59) and growth cones (PCC 0.39±0.05; MOC 0.53±0.04; n = 6, N = 49) a partial overlap was observed (Fig. 2A, C). When motoneurons were cultured on laminin-221/211, a condition which leads to maturation of presynaptic terminals [15], [45], [46], neither the subcellular distribution of hnRNP R nor the degree of codistribution and correlation of Smn and hnRNP R changed significantly in motoneuron cell bodies (PCC 0.53±0.02, P = 0.0582; MOC 0.67±0.02, P = 0.0814; n = 6, N = 51), axons (PCC 0.35±0.05, P = 0.1172; MOC 0.50±0.03, P = 0.0617; n = 6, N = 50) or axonal growth cones (PCC 0.31±0.05, P = 0.1004; MOC 0.48±0.02, P = 0.1060; n = 6, N = 43) (Fig. 2B, C). Similar results were obtained with an independent N-terminal hnRNP R antibody with respect to codistribution of Smn and hnRNP R in these isolated motoneurons (Fig. S1D). To further characterize the colocalization of Smn and hnRNP R immunofluorescence we used ImageJ for a colocalization test calculating random PCC values which reflect a computational non-related random overlap of two signals. Each colocalization analysis of hnRNP R and Smn produced a PCC value which was significantly higher than the corresponding randomized value. Thus, a non-random codistribution of hnRNP R and Smn can be assumed (for more details see material and methods).

We then examined whether the subcellular location of hnRNP R and the colocalization and correlation of Smn and hnRNP R are regulated over time when motoneurons grow and differentiate *in* *vitro*. We cultured motoneurons on laminin-111 and determined the localization of hnRNP R and the degree of overlap with Smn from day 1 to day 7. Previous analyses have demonstrated that axon elongation in isolated motoneurons from E13.5 mouse embryos is highest around 4DIV, corresponding to day 18 of embryonic development [15]. Therefore, we chose 3DIV (Fig. 2D) and 7DIV (Fig. 2E) as time points for quantitative analysis. Surprisingly, the subcellular distribution of hnRNP R changed between 3DIV and 7DIV in motoneuron cell bodies. In comparison to 3DIV (n = 5, N = 37) the relative ratio of cytosolic versus nuclear hnRNP R immunoreactivity was significantly increased by 63% (P = 0.0173, n = 5, N = 46) at 7DIV (Fig. 2D, E). This relatively higher number of hnRNP R-positive granules in the cytoplasm was accompanied by enhanced codistribution and correlation of hnRNP R and Smn, as detected by colocalization analysis in motoneuron cell bodies at 7DIV versus 3DIV (PCC 15%, P = 0.0112; MOC 10%, P = 0.0086). Similar alterations were also observed in axonal growth cones (PCC 18%, P = 0.0467; MOC 8%, P = 0.1565), but not in axons (PCC 7%, P = 0.1504; MOC 5%, P = 0.1449) (Fig. 2D–F). This shift in location and colocalization coincides with rapid axon extension starting at 4DIV. Interestingly, defects in axon elongation in Smn- [15] or hnRNP R- [29] deficient motoneurons cultured under similar conditions are most profound between 4DIV and 7DIV indicating an important contribution of Smn to the subcellular distribution of hnRNP R and by this way possibly to axonal outgrowth.

### The interaction of Smn and hnRNP R varies between different cellular compartments

**Figure 3:**
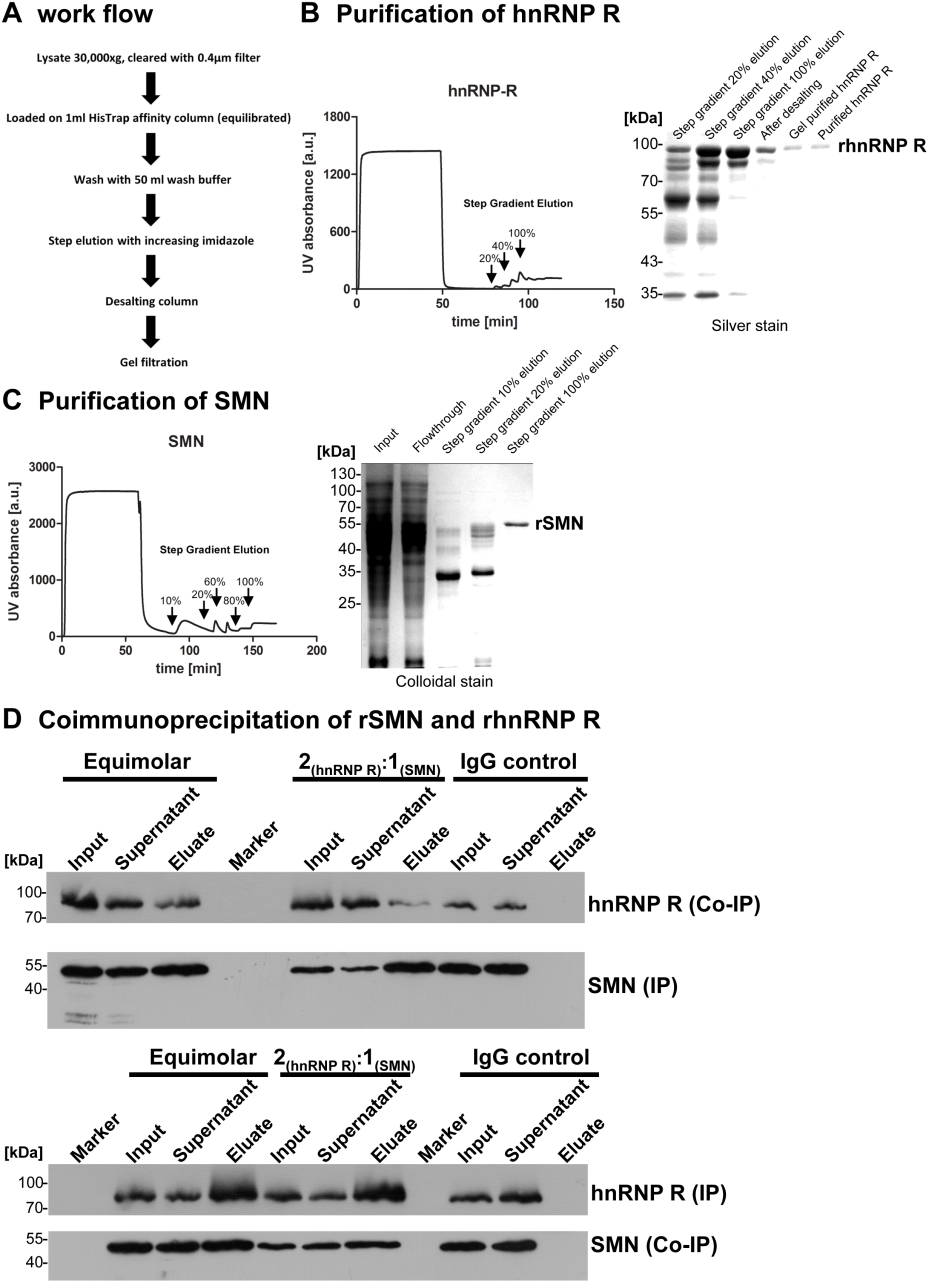
Direct interaction of hnRNP R and SMN. (A) Purification scheme of recombinant hnRNP R and SMN expressed as His-tagged proteins in *E. coli* strain BL21. (B) Affinity purification profile on a fast protein liquid chromatography (FPLC) of hnRNP R and SDS-PAGE of recombinant hnRNP R purification steps visualized by silver staining. (C) Affinity purification profile on a FPLC of SMN and SDS-PAGE of recombinant SMN purification steps visualized by colloidal staining. (D) Coimmunoprecipitation of recombinant SMN and hnRNP R.

In a further step we investigated whether the interaction between Smn and hnRNP R is direct (Fig. 3) by expressing recombinant hnRNP R and SMN in *E. coli* purifying both proteins to homogeneity (Fig. 3A–C). This allowed us to test the interaction of hnRNP R and SMN in the absence of other proteins. Both proteins could be coimmunoprecipitated when equimolar concentrations were analyzed indicating that Smn and hnRNP R interact directly in the absence of other protein binding partners or RNA (Fig. 3D). HnRNPs are known to form homomeric interactions [47]. In order to test whether the formation of hnRNP R dimers influences binding to Smn we doubled the amount of recombinant hnRNP R in this assay. When SMN was now pulled down, less hnRNP R was coimmunoprecipitated and *vice versa*, whereas the efficacy of the immunoprecipitation itself was comparable between both experimental conditions (Fig. 3D). The IgG control was negative thus validating the specificity of the detected interaction (Fig. 3D).

**Figure 4:**
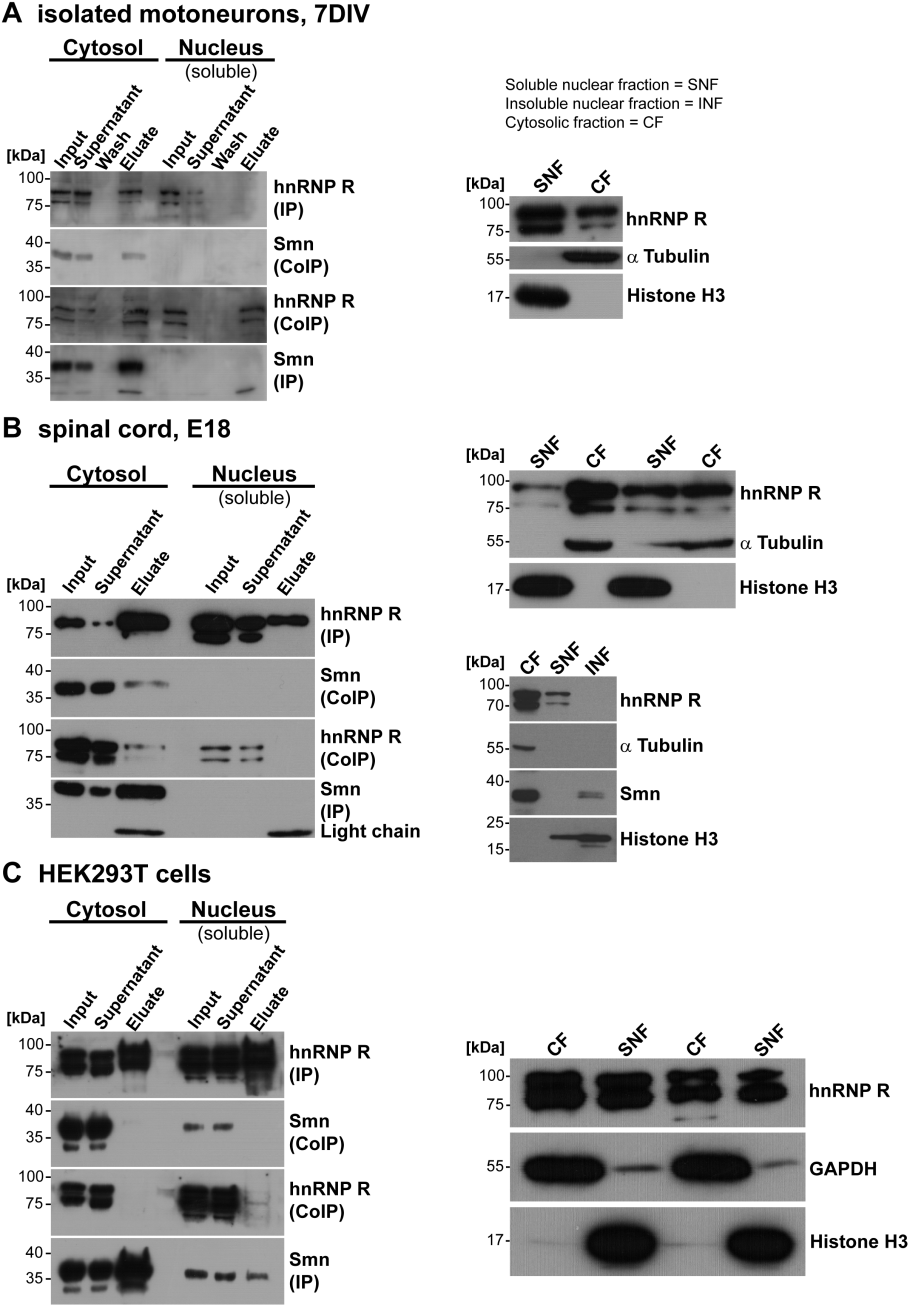
Coimmunoprecipitation of Smn and hnRNP R in primary motoneurons and native spinal cord. (A) 1 000 000 primary motoneurons were cultured for 7DIV on laminin-111. Cytosolic and soluble nuclear fractions were subjected to a pull-down with either Smn or hnRNP R antibodies, respectively. Coprecipitation of hnRNP R or Smn, respectively, was determined revealing an interaction of Smn and hnRNP R, particularly in the cytosolic fraction of embryonic mouse motoneurons (eluate lane). Smn was not detectable in the soluble nuclear fraction of motoneurons. HnRNP R was found both in nuclear and cytosolic extracts. For immunoprecipitation experiments a C-terminal antibody directed against hnRNP R (Abcam) was used [29]. Supernatants still contained some Smn or hnRNP R protein, respectively, suggesting that the interaction appears not to be exclusive as demonstrated by immunofluorescence colocalization analysis. No signal was obtained in the washing solution. Successful fractionation was controlled by α tubulin (cytosol) and histone H3 (nucleus) (right panel). (B) Fractionation of spinal cord tissue from E18 mouse embryos revealed a similar result as shown in (A). In the cytosolic fraction hnRNP R IP pulled-down Smn protein and *vice versa*. Nuclear Smn was not detected in the soluble, but in the corresponding insoluble nuclear fraction (right panel, lower blot). In contrast, nuclear hnRNP R was not found in the insoluble nuclear fraction. Cytosolic and nuclear extracts were validated by α tubulin and histone H3. (C) HEK293T cells were cultured and cytosolic and soluble nuclear fractions were prepared. Smn and hnRNP R were detected in cytosolic extracts as well as in soluble nuclear fractions. The pull down of Smn and hnRNP R, respectively, was successful (eluate lane, IP), but hnRNP R or Smn, respectively, could not be coprecipitated, neither from cytosolic nor from nuclear extracts. Successful fractionation was verified by GAPDH (cytosolic) and histone H3 (nucleus) (right panel).

We proceeded to examine whether the interaction of hnRNP R and Smn differs between cellular compartments (Fig. 4) using cytosolic and nuclear fractions from isolated motoneurons (Fig. 4A), E18 spinal cord (Fig. 4B) and HEK293T cells (Fig. 4C). Motoneurons were cultured for 7DIV on laminin-111 since the relative proportion of cytosolic hnRNP R and the degree of overlap with Smn protein was highest at this time point as described above. Antibodies against histone H3 were used as marker for the nuclear fraction, and antibodies against α tubulin and GAPDH for the cytosolic fraction (Fig. 4A–C, right panels). HnRNP R was found both in the soluble nuclear and in the cytosolic fraction. Intriguingly, interaction of Smn and hnRNP R was predominantly detected in cytosolic compartments of cultured motoneurons (Fig. 4A) and spinal cord extracts (Fig. 4B). Pulldown of hnRNP R coprecipitated Smn and *vice versa*.

Smn was not detected in the soluble nuclear fraction (Fig. 4A, B, input lane), but in the corresponding insoluble nuclear fraction (Fig. 4B, right panel, lower blot), showing two bands, which may reflect phosphorylation. Interestingly, the phosphorylation state of Smn has been described to determine its nuclear localization to Gems and Cajal bodies [48]–[52]. In contrast, hnRNP R levels in this insoluble nuclear fraction are below detection limit indicating that hnRNP R and Smn are present in distinct compartments within the nucleus, which argues against a nuclear interaction.

HEK293T cells differed from isolated motoneurons and spinal cord extracts by showing detectable nuclear Smn levels in soluble fractions together with hnRNP R (Fig. 4C). In these cells, no interaction of Smn and hnRNP R was found by coimmunprecipitation, neither in the cytosolic nor in the soluble nuclear fraction indicating that the interaction of Smn and hnRNP R differs between neuronal and nonneuronal cells (Fig. 4C).

### Localization of Smn and hnRNP R in spinal motoneurons and neuromuscular endplates

**Figure 5:**
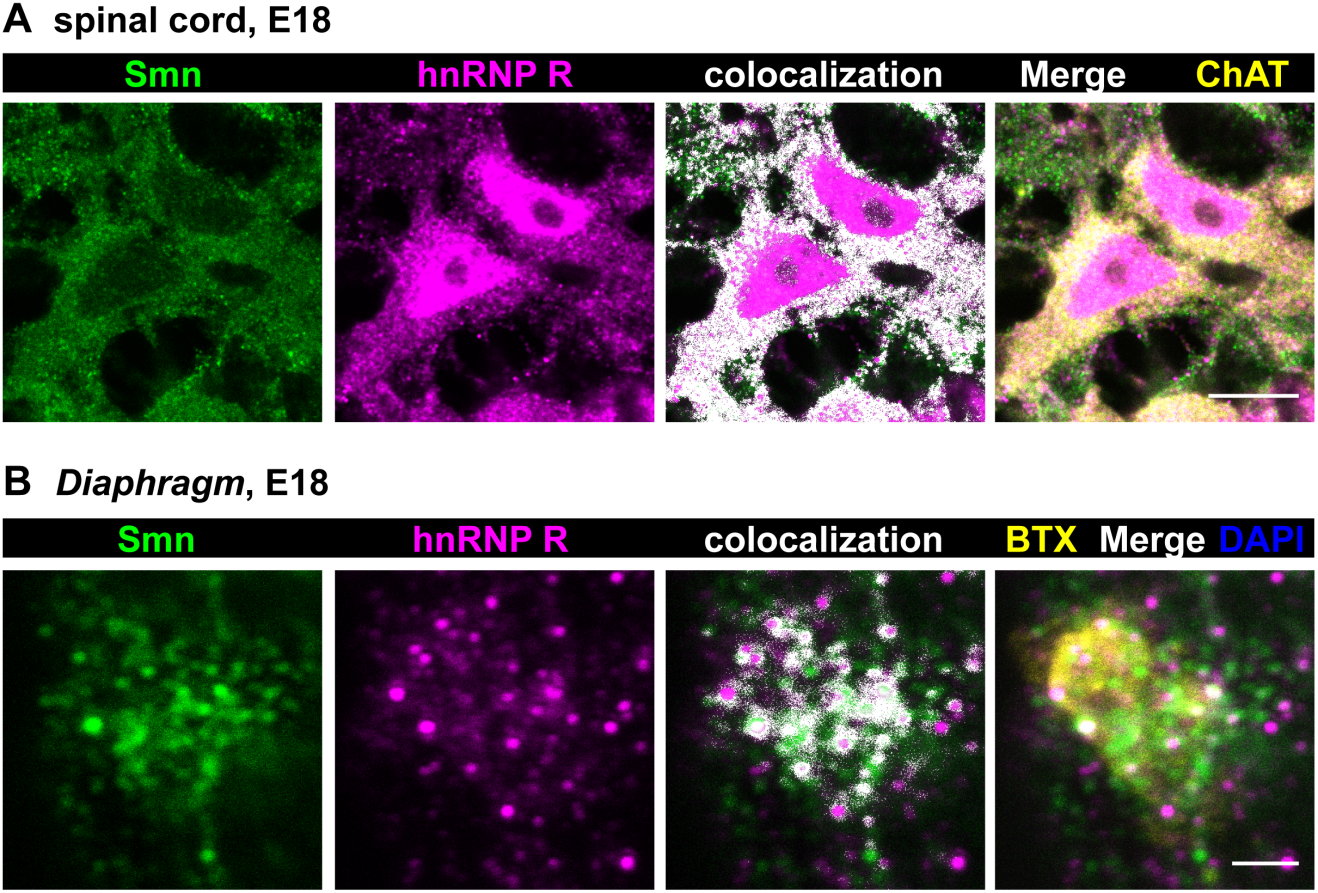
Colocalization of Smn and hnRNP R *in* *vivo* in E18 motoneurons and axon terminals. (A) Representative cross section from E18 spinal cord stained against Smn, hnRNP R and ChAT (scale bar: 10 µm). Superimposed colocalizing points are highlighted in white. Smn signals were mainly found in the cytosol, with very few positive spots in the nuclei. HnRNP R immunoreactivity was observed in the nucleus and in the cytosol. Colocalization of Smn and hnRNP R was detected in the cytosol, especially in axonal initiation segments (PCC 0.27±0.03, MOC 0.81±0.01, N = 8). (B) Whole mount preparations from *Diaphragm* muscles from E18 mouse embryos stained against Smn, hnRNP R, ω-BTX and DAPI (scale bar: 2 µm). Both Smn and hnRNP R immunoreactivity were detected at these defined sites showing partial overlap (PCC 0.24±0.04, MOC 0.54±0.02, N = 6).

Based on these results we studied distribution and colocalization of Smn and hnRNP R in spinal cord cross sections from E18 mouse embryos (Fig. 5A) which correlates with the developmental stage of primary motoneurons isolated at E13.5 and cultured for 5DIV. Motoneurons were identified by choline acetyltransferase (ChAT) staining. Again, Smn immunoreactivity was mostly found in the cytosol and in proximal axonal processes, whereas nuclei appeared relatively spared revealing only distinct Gem-like immunoreactive structures. In contrast, hnRNP R was detected both in the nucleus and in the cytosol. In particular, perinuclear cytoplasm and proximal axons showed an overlap of hnRNP R and Smn signals (PCC 0.27±0.03; MOC 0.81±0.01; N = 8) (Fig. 5A) which is similar to the data obtained by immunofluorescence in isolated embryonic motoneurons (see Fig. 2) and Western blot analyses of coimmunoprecipitation from cytosolic fractions (see Fig. 3).

In order to address whether Smn and hnRNP R are also present in axon terminals *in* *vivo* we examined neuromuscular endplates in the *Diaphragm* from 18-day old mouse embryos (Fig. 5B). Motor endplates in whole mount preparations of the *Diaphragm* were identified by ω-bungarotoxin (BTX) staining of postsynaptic acetylcholine receptors. At this site, Smn- and hnRNP R-positive signals were detected with partially colocalizing points (PCC 0.24±0.04; MOC 0.54±0.02; N = 6).

**Figure 6:**
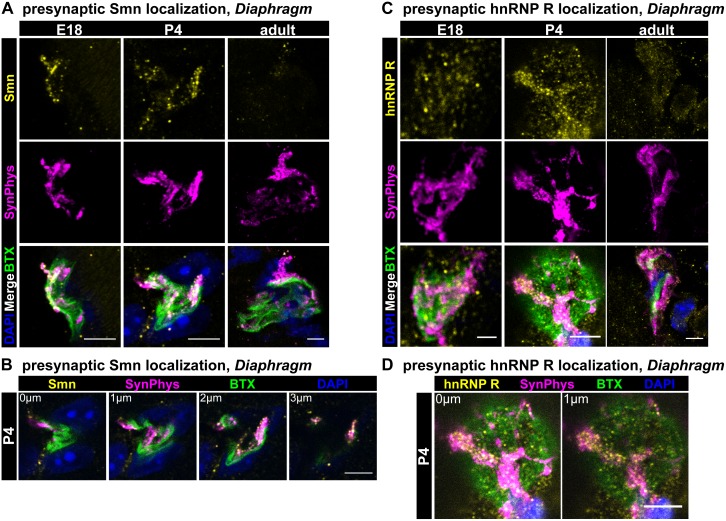
Localization of Smn and hnRNP R at neuromuscular junctions from E18, P4 and adult *Diaphragm*. Whole mount preparations from *Diaphragm* muscles from developmental (E18) (A, C, left panels), postnatal (P4) (A, C, middle panels) and adult (3 months) (A, C, right panels) stages were performed (scale bar: 2 µm (C, left panel), 5 µm). (A) Muscles were stained against ω-BTX, SynPhys, DAPI and Smn protein. (A, left panel) At E18 Smn was highly enriched in presynaptic structures identified by SynPhys immunoreactivity. Few spots appeared in postsynaptic nuclei. (A, middle panel) Smn-positive signals were also detected in P4 motor endplates coresiding with SynPhys staining. Postsynaptic nuclei showed faint Smn immunoreactivity. (A, right panel) In 3 month old mice (adult stage) less Smn-positive signals were noticed as described before [53], [56]. The few immunoreactive particles were predominantly located in presynaptic structures visualized by SynPhys staining. (B) Single optical slices of the P4 neuromuscular synapse highlighted the co-occurring SynPhys and Smn signals (scale bar: 5 µm). (C) Muscles were stained against ω-BTX, SynPhys, DAPI and hnRNP R. HnRNP R was codistributed with SynPhys in presynaptic compartments at E18 (left panel), P4 (middle panel) and adult stage (right panel). HnRNP R was also detected in postsynaptic structures revealing stronger immunoreactivity at these sites in comparison to Smn. (D) Single optical slices of the P4 motor endplate emphasized the presynaptic localization of hnRNP R (scale bar: 5 µm).

To characterize the localization of Smn and hnRNP R at neuromuscular junctions in more detail, confocal microscopy at different developmental stages was performed with synaptophysin (SynPhys) as a marker for presynaptic terminals (Fig. 6). Postsynaptic nuclei were visualized by DAPI staining. At E18, Smn was strongly enriched in presynaptic compartments (Fig. 6A, left panel). Smn-positive signals were also detected in presynaptic terminals at postnatal day 4 (Fig. 6A, middle panel, 6B, Fig. S2A) and in the adult (Fig. 6A, right panel). However, levels of Smn immunoreactivity were lower at the latter stage, which corresponds to decreased Smn expression in spinal cord of adult mice [53]. At these analyzed neuromuscular junctions postsynaptic nuclei and the postsynaptic space labeled by BTX contained few Smn-positive signals at any developmental stage which confirms muscular expression and localization [54]–[58]. We also performed cryostat sections of ventral roots of the gastrocnemic muscle of adult mice and observed both Smn- and hnRNP R-positive signals in motor axons of sciatic nerves at this stage *in* *vivo* (Fig. S2C).

HnRNP R protein was mainly colocalized with synaptophysin in presynaptic terminals in the *Diaphragm* at E18 (Fig. 6C, left panel). In addition, hnRNP R was detected in postsynaptic structures. Similar findings were obtained at P4 (Fig. 6C, middle panel, 6D, Fig. S2B) and in the adult (Fig. 6C, right panel). In the adult, hnRNP R immunoreactivity appeared reduced in presynaptic terminals reflecting decreased hnRNP R expression in motoneurons during postnatal development [18]. As a control, preabsorption with recombinant hnRNP R highly depleted hnRNP R immunoreactivity implying that the signals detected by ICN 1-18 were also specific *in* *vivo* (Fig. S3).

### Reduced Smn immunoreactivity at neuromuscular junctions of a SMA type I mouse model

**Figure 7:**
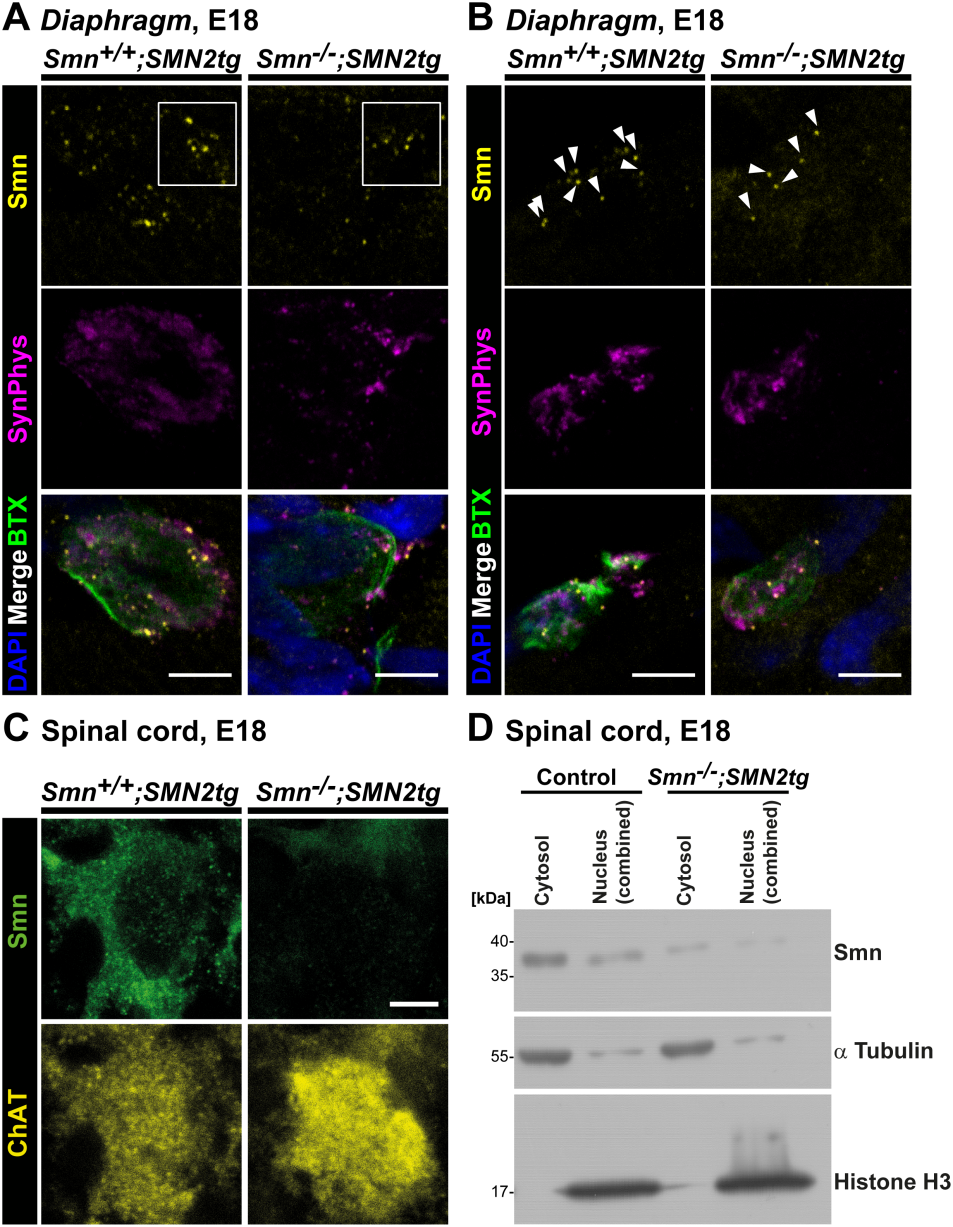
Smn deficiency in SMA type I axon terminals *in* *vivo.* (A, B) Representative motor endplates from E18 *Smn^+/+^; SMN2tg* and *Smn^−/−^; SMN2tg Diaphragm* stained against Smn and SynPhys. Acetylcholine receptors (AChR) and postsynaptic nuclei were visualized by ω-BTX and DAPI, respectively (scale bar: 5 µm). In (A) Smn deficiency is visible by highly reduced immunoreactive signals, as highlighted in the white box, whereas in (B) the number of Smn particles per NMJ is decreased in SMA type I motor endplates, as indicated by white arrowheads. (A, B) In SMA type I axon terminals (n = 3, N = 32) mean Smn signal intensity was significantly reduced (0.43±0.09, P = 0.0220, t = 6.629, DF = 2) in comparison to control motor endplates (set as ‘1’, n = 3, N = 43), whereas SynPhys signals (*Smn^−/−^; SMN2tg* 1.15±0.19, P = 0.5221, t = 0.7694, DF = 2) and the size of the presynaptic compartment (Control 49.48±13.94 µm^2^; *Smn^−/−^; SMN2tg* 36.56±7.464; P = 0.4596, t = 0.8174, DF = 4) were comparable. (C) Representative images from E18 *Smn^+/+^; SMN2tg* and *Smn^−/−^; SMN2tg* spinal cord cross sections immunolabeled with Smn and ChAT. Quantitative analysis revealed a significant decrease in cytosolic Smn immunoreactivity in SMA type I motoneurons in comparison to *Smn^+/+^; SMN2tg* cells (*Smn^+/+^; SMN2tg* set as ‘1’, n = 6, N = 107; *Smn^−/−^; SMN2tg* 0.46±0.05, n = 6, N = 85; P<0.0001, t = 11.23, DF = 5). ChAT signal intensity was not statistically affected (*Smn^−/−^; SMN2tg* 0.83±0.21; P = 0.4638, t = 0.7928, DF = 5). (D) Representative Western Blot with cytosolic and nuclear fractions from E18 control and *Smn^−/−^; SMN2tg* spinal cord extracts. Histone H3 and α tubulin were used as markers for nuclear and cytosolic fractions, respectively, and as standardization proteins for quantitative analysis. In SMA type I spinal cord extracts cytosolic and nuclear Smn were significantly reduced by 64% (0.36±0.08, N = 10, P<0.0001, t = 8.480, DF = 9) and 86% (0.14±0.03, N = 10, P<0.0001, t = 26.39, DF = 9), respectively, in comparison to *Smn^+/+^; SMN2tg* extracts (set as ‘1’, N = 10).

To validate the specificity of the observed presynaptic Smn staining *in* *vivo,* whole mount preparations from three E18 *Smn^−/−^; SMN2tg* mouse *Diaphragms* were analyzed and compared with controls (Fig. 7), revealing a significant reduction of the mean Smn signal intensity of 57% in SMA type I NMJs (0.43±0.09, P = 0.0220, n = 3, N = 32) in comparison to control samples (n = 3, N = 43), whereas neither the size of the presynaptic compartment nor SynPhys signal intensities were significantly altered at this developmental stage (Fig. 7A, B). We also investigated cytosolic Smn immunoreactivity in the corresponding E18 *Smn^−/−^; SMN2tg* (n = 6, N = 85) motoneuron cell bodies in spinal cord cross sections, detecting a significant decrease of 54% (0.46±0.05, P<0.0001) in comparison to *Smn^+/+^; SMN2tg* cells (n = 6, N = 107) (Fig. 7C). These two results were at variance with previous studies reporting profound loss of Smn protein in the range of 80% in brain extracts from these mice [59]. Therefore, we analyzed cytosolic and nuclear fractions from four E18 SMA type I spinal cords and corresponding control tissue in order to obtain more robust biochemical data and to validate the aforementioned immunohistochemical quantitative analysis (Fig. 7D). Smn protein levels were significantly reduced by 86% (0.14±0.03, n = 10, P<0.0001) in nuclear and by 64% (0.36±0.08, n = 10, P<0.0001) in cytosolic fractions of *Smn^−/−^; SMN2tg* spinal cord, respectively. With respect to the underlying biological variances derived from independent embryos and litters *in* *vivo* we concluded from these data that the differences determined by immunohistochemistry were in line with the reduction of cytosolic Smn protein quantified by biochemical analysis, thus confirming the specificity of the applied Smn antibody also *in* *vivo*.

## Discussion

Since the discovery of *SMN* mutations as cause of SMA multiple efforts have been made in elucidating the role of the corresponding protein particularly in motoneuron development and maintenance. Whilst SMN has a central cellular role in the assembly of spliceosomal snRNPs (reviewed in [38]) it is now becoming increasingly clear that SMN also interacts with a number of RNA-binding proteins such as FMRP [30], KSRP [60], hnRNP R and Q [18], [19], TDP-43 [61], FUS [31], IMP1 [62] and HuD [36], [63], [64]. In this study we provide evidence that Smn colocalizes and interacts with hnRNP R in distinct subcellular compartments of motoneurons. Beside the already known location in nucleus and cytosol both proteins are present in axon terminals *in* *vivo* at embryonic and postnatal stages providing additional weight to the hypothesis that Smn, together with hnRNP R and possibly also other mRNA-binding proteins, contributes significantly to maturation and function of neuromuscular synapses by direct local action in the presynaptic compartment [4], [8], [12]–[14].

HnRNP R has been identified as an interaction partner of Smn [18]. Furthermore, hnRNP R binds to U-rich sequences within the 3′UTR of β-actin mRNA [19], [29] and participates in the translocation of this mRNA into axons and axon terminals [19]. Accordingly, loss of either Smn or hnRNP R reduces axon growth of isolated mouse motoneurons [19], [29]. Smn-deficient motoneurons exhibit defects in the actin cytoskeleton in axonal growth cones resulting in impaired maturation and differentiation of these specialized structures to presynaptic terminals at neuromuscular endplates [15], [19], [65]. This correlates with defective translocation of Ca_v_2.2 calcium channels and eventually other transmembrane proteins to the surface, preventing calcium influx and the recognition of essential differentiation signals provided by direct interaction of Ca_v_ α subunits and β2 laminin chains [15], [46], [66]. In line with these observations, depletion of Smn or hnRNP R in zebra fish leads to comparable phenotypes with respect to truncated motor axons and aberrant branching in peripheral regions pointing to a common functional pathway also *in* *vivo* [29], [37].

Recently, Smn has been visualized in spinal motoneuron cell bodies *in* *vivo* [8], [32], [67], [68], whereas its presence in the presynaptic compartment of neuromuscular junctions, particularly of postnatal mice, at least to our knowledge, has not been reported yet. Previous attempts to detect SMN in these structures have rather revealed a codistribution with postsynaptic marker BTX than with presynaptic markers SynPhys or neurofilament (NF) [7]. Notably, Smn immunoreactivity has also been detected in skeletal muscle [56], [57], which complicates reliable visualization of presynaptic Smn. In this study we chose the *Diaphragm* to perform immunohistochemistry at neuromuscular synapses to ensure controlled orientation due to the defined anatomy of the *Diaphragm*. Furthermore, we applied IgG_1_ mouse antibodies for immunodetection reducing the probability of false-positive signals derived from unspecific binding of the applied mouse monoclonal SMN antibody to endogenous mouse IgG antibodies and homologous adhesion molecules. Smn expression is known to decrease in motoneurons at later postnatal stages, which makes it difficult to detect Smn protein in sections of spinal cord, motor nerves and at neuromuscular endplates [53]. Nevertheless, we were able to visualize Smn in presynaptic motor nerve terminals particularly of E18 and P4 neuromuscular junctions in addition to the already reported postsynaptic intramuscular localization [7], [56], [57].

Smn and hnRNP R are partially colocalizing in axons and axon terminals and also in the perinuclear region within the soma of motoneurons. Since both hnRNP R and Smn have numerous interaction partners with various functions, this spatial distribution and correlation is not surprising and indicates that dynamic interactions of Smn, hnRNP R and other RNA binding proteins could take place in axons and axonal compartments which need to be investigated in more detail. This hypothesis is supported by the observation that the axonal and presynaptic colocalization of hnRNP R with Smn changes over time. The highest degree of overlap is observed when axon elongation and presynaptic differentiation occur [15]. This dynamic change in codistribution and the relatively high levels of these proteins in cytosolic structures during this period could correspond to the *in* *vitro* deficits in axon growth observed in Smn- [15], [19] and hnRNP R-deficient [29] motoneurons. In order to corroborate this result, coimmunoprecipitation experiments were performed with recombinant and purified Smn and hnRNP R, and also with isolated motoneurons, spinal cord extracts and non-neuronal cells. These experiments revealed a direct interaction of hnRNP R and Smn predominantly in the cytosol of motoneurons. In HEK293T cells, Smn and hnRNP R could not be coimmunoprecipiated, neither from nuclear nor from cytosolic extracts thus pointing to differences between neuronal and other cell populations.

Recently, it has been demonstrated that mutant FUS sequesters axonal Smn [69], disturbs snRNP localization [70], reduces the number of Gems [71] and develops synaptic defects at neuromuscular junctions [72], [73], thus establishing a potential correlation between ALS and SMA [74]. Similar results were reported for TDP-43 [33]. Mutant TDP-43 reveals impaired transport of cytoplasmic mRNP granules [75]. Notably, axonal transport deficits have also been identified in SMNΔ7 mice [76]. In our study, shRNA-mediated hnRNP R depletion did not interfere with Smn expression or the number of Gems per nucleus. Equally, Smn depletion did not alter hnRNP R protein levels in motoneurons, indicating that these two proteins are not major regulators of each other at the levels of transcription and early pre-mRNA processing. This appears different with other members of the hnRNP family that control Smn levels at the pre-mRNA processing stage [77]–[83]. Thus, cytosolic hnRNP R that is bound to Smn could exert unique functions in comparison to nuclear hnRNP R and other members of the hnRNP family.

Nuclear and perinuclear Smn could also affect the assembly and axonal transport of protein/RNA-containing particles, and this process could potentially contribute to SMA pathology. Recent data have shown that Smn mediates the axonal localization of IMP-1 [62] and the trafficking of cpg15 mRNA via binding to HuD [64], additionally regulating local translation [63], [84]. In line with these findings are reports stating that mutant hnRNP A2B1 and A1 are incorporated into stress granules resulting in aberrant cytoplasmic inclusions, which possibly impairs their axonal function [85]. Furthermore, more than 200 mRNAs associated with SMN have been identified in differentiated NSC-34 cells with 30% revealing an SMN-dependent axonal localization [86]. Using RNA-seq techniques, cell-specific mRNA transcriptome changes have been described that affect NMJ formation and maintenance [87] and it appears logical that these alterations can be assigned to axonal and/or somatodendritic compartments. Taken together, a similar functional relationship of Smn and hnRNP R, i.e. a Smn-dependent axonal translocation of hnRNP R and hnRNP R-bound mRNAs, may therefore be a legitimate assumption.

## Conclusion

Biochemical and immunohistochemical analyses performed in this study provide evidence of a direct interaction of Smn and hnRNP R in spinal motoneurons *in* *vitro* and *in* *vivo*, predominantly in the cytosolic compartment. Both proteins are present in axons and axon terminals of motoneurons *in* *vitro* and *in* *vivo*. We hypothesize that axonal and presynaptic Smn and hnRNP R proteins are not involved in U snRNP assembly, but exert a non-canonical function which contributes to differentiation and maintenance of neuromuscular endplates.

## Materials and Methods

### Animals and ethics statement

C57Bl/6, CD-1 and SMA type I transgenic mice [59] were kept at the animal facilities of the Institute for Clinical Neurobiology at the University hospital of Wuerzburg providing controlled conditions such as food and water in abundant supply, 20–22°C, a 12 hours light/dark cycle, and 55–65% humidity, respectively. Each experiment was performed strictly following the regulations on animal protection of the German federal law, the Association for Assessment and Accreditation of Laboratory Animal Care and of the University of Wuerzburg, in agreement with and under control of the local veterinary authority and Committee on the Ethics of Animal Experiments, i.e. Regierung von Unterfranken, Wuerzburg (License numbers 566/200-244/13 and 55.2-2531.01-08/14).

This study was approved by the local veterinary authority (Veterinaeramt der Stadt Wuerzburg) and Committee on the Ethics of Animal Experiments, i.e. Regierung von Unterfranken, Wuerzburg (License numbers 566/200-244/13 and 55.2-2531.01-08/14).

### Isolation and culture of primary embryonic mouse motoneurons

Spinal cord tissue from E13.5 mouse embryos was isolated and motoneurons were enriched via p75-panning as described previously [88]. In brief, lumbar spinal cord was dissected and processed thoroughly by removing dorsal root ganglia (DRGs) and meninges. After digestion with 0.1% trypsin (Worthington) motoneurons were enriched by incubation with anti-p75 antibody-coated (MLR2, Abcam) cell culture dishes. Cells were counted and plated on cell culture dishes or glass cover slips which had been coated with laminin-111 or laminin-221/211, respectively. Motoneurons were cultured in the presence of 10 ng/ml BDNF and CNTF for 5DIV or 7DIV, respectively, at 37°C in a 5% CO_2_ atmosphere. Motoneuron medium, comprising Neurobasal Medium (Gibco), 2% horse serum, 500 µM GlutaMAX-I (Gibco) and B27 (1∶50, Gibco), was changed at 1DIV and then every second day.

Lentiviral knockdown experiments were performed by incubation of motoneuron directly before plating with either control or knockdown viruses, respectively, for 8 min at RT. Infected cells were identified by GFP reporter expression from lentiviral constructs.

### Immunocytochemical analysis of embryonic mouse motoneurons

Cells were washed with warm PBS (PAA Laboratories, pH 7.4) to remove serum and debris, and fixed with 4% paraformaldehyde (PFA) for 15 min at RT. Treatment with 0.3% TritonX for 20 min at RT ensured decent antibody penetration of the nuclei. Unspecific binding of antibodies was reduced to a minimum by blocking with either 10% BSA or serum of the species of the secondary antibody, i.e. goat or donkey serum, respectively. Primary antibodies were applied overnight at 4°C. Cells were washed thoroughly and incubated with appropriate fluorescent secondary antibodies. Nuclei were counterstained with DAPI. Coverslips were embedded with Mowiol (Sigma-Aldrich, 10852) and imaged subsequently.

The following primary and secondary antibodies were used in this study: monoclonal mouse anti-SMN (1∶250, BD Biosciences, 610646), polyclonal rabbit anti-hnRNP R (1∶250, Sigma HPA026092; 1∶2000, polypeptide antiserum aa1-18, ICN, Wuerzburg), polyclonal guinea pig anti-Synaptophysin (1∶600, Synaptic Systems), polyclonal chicken anti-Neurofilament (heavy chain) (1∶5000, Millipore AB5539), goat anti-mouse (H+L) IgG_1_ (Cy5, 1∶500, Abcam ab136127), donkey anti-rabbit (H+L) IgG (Cy3, 1∶700, Jackson Immunoresearch 711-165-152), donkey anti-guinea pig (H+L) IgG (Cy2, 1∶400; Dianova 706-225-148) and donkey anti-chicken (H+L) IgG (DyLight 649, 1∶500, Jackson Immunoresearch 703-495-155).

### Knockdown of Smn and hnRNP R via lentiviral shRNA in embryonic motoneurons

Viruses were produced according to the manufacturer’s instructions expressing either shRNA against Smn or hnRNP R, respectively, or a GFP-reporter gene as internal control. The knockdown vector for hnRNP R and Smn was generated by cloning hnRNP R (5′-GATGCTCTCAGGGAGTTTAAT-3′) and Smn (5′-GAAGAATGCCACAACTCCC-3′) shRNA sequence into the pSIH-H1 shRNA vector (System Bioscience). HEK293T cells were used to generate viruses as described previously [89], [90].

### Data analyses and statistics

At least three independent experiments were performed for statistical analysis. Data are expressed as mean ± standard error of the mean (SEM). ‘N’ indicates the total number of analyzed specimens, e.g. NMJs, axons, growth cones or motoneuron cell bodies, and ‘n’ the number of individual specimens, e.g. different embryos from different litters, different wells from independent cultures or different object slides and technical Western Blot replicates from different embryos, which were statistically scored. For comparison of two groups unpaired (Fig. 1E, Fig. 7A, B) or paired (Fig. 2, Fig. S1C) student’s t-test, or one sample t-test (Fig. 2C, D, Fig. 7A–D, Fig. S1C) was used, respectively. For comparison of three groups (Fig. 1B) ‘Repeated Measures ANOVA’ with post-hoc Bonferroni multiple comparison was applied.

For statistical analyses the GraphPad Prism 4.02 software (SanDiego, CA) was used. Fluorescence intensities were measured as mean gray values per stained area and displayed as arbitrary units, based on quantum levels (QL) per pixel, using the Leica LAS AF LITE Software. Signal intensities were determined from raw images for each optical slice by subtracting background intensities from the measured immunoreactive signals. To determine the relative Smn fluorescence intensity of motor endplates, average intensity stacks were created from confocal data sets, and the mean signal intensity of all Smn particles of one analyzed neuromuscular junction was scored. For calculating the ratio between cytosolic and nuclear compartments the sizes of the determined regions of interests were taken into account. Values of consistent control groups and relative values of control groups were standardized to ‘1’ and data from different experiments were combined when control values were comparable to each other.

### Image acquisition and processing

For image acquisition the Leica TCS SP2 and SP5 confocal systems were used, as well as the Olympus Fluo ViewTM FV1000 microscope. For intensity measurement identical settings were applied, i.e. objective, magnification, laser intensity and photomultiplier. Final processing of all images was performed with Image-J (MacBiophotonics), Photoshop 7.0 (Adobe) and Illustrator CS5 (Adobe). The average intensity stack function was used in figure 1B, E, and S1C, and the maximum intensity stack function in figure 1C (upper panel, i.e. cell body), 5B, 6A, C (middle and right panel), 7A, B, S2A–C and S3A, B. In figure 6 and figure S2A, B postsynaptic motor endplate staining by BTX was smoothened for better visualization.

Brightness and contrast were enhanced in the following images for better visualization:

**Figure 6A** **(8bit) (left panel):** BTX (MIN20), DAPI (MIN10), SynPhys (MIN20), Smn (MIN10)
**Figure 6A** **(8bit) (middle panel):** BTX (MIN30), DAPI (MIN20), SynPhys (MIN20), Smn (MIN20)
**Figure 6A** **(8bit) (right panel):** BTX (MIN20, MAX175), DAPI (MIN20), SynPhys (MIN10 MAX150), Smn (MIN10)
**Figure 6C** **(8bit) (left panel):** BTX (MIN20), SynPhys (MIN20), hnRNP R (MIN20)
**Figure 6C** **(12bit) (middle panel):** BTX (MIN200 MAX2500), DAPI (MIN200), SynPhys (MIN400), hnRNP R (MIN350)
**Figure 6C** **(8bit) (right panel):** BTX (MIN20), DAPI (MIN20), SynPhys (MIN20), hnRNP R (MIN25)
**Figure 7A, B** **(8bit):** BTX (MIN20), DAPI (MIN10), SynPhys (MIN5), Smn (MIN20)
**Figure 7C** **(12bit):** ChAT (MIN200 MAX 3500), Smn (MIN200 MAX 2000)
**Figure S3A, B (8bit):** BTX (MIN10), DAPI (MIN10), SynPhys (MIN10), hnRNP R (MIN20)

### Colocalization analysis

Colocalization was analyzed using the Pearson’s correlation coefficient (PCC) and the Manders Overlap Coefficient (MOC) plugin of ImageJ. MOC measures the percentage of overlap of two signals computationally standardizing size and intensity and excluding ‘zero’ pixels. Thus, co-occurrence of individual fluorophores is determined. Perfectly colocalizing points within the spatial resolution of the used objective, magnification and microscope are rated ‘1’. In contrast, PCC is applied to quantify the correlation between individual fluorophores taking their intensities into consideration. To exclude a ‘random colocalization’ of Smn and hnRNP R we used ImageJ for a colocalization test with Fay randomization which compares and validates the PCC of the ‘real’ image against 25 ‘randomly created’ images generated by repeatedly shifting pixels of one of the color channels:

**Figure 2A, C** **(5DIV, Laminin)**: Cell body (PCC_real_ = 0.59, PCC_random_ = 0.55), axon (PCC_real_ = 0.42, PCC_random_ = 0.22), growth cone (PCC_real_ = 0.39, PCC_random_ = 0.25)
**Figure 2B, C** **(5DIV, Laminin-221/211)**: Cell body (PCC_real_ = 0.53, PCC_random_ = 0.50), axon (PCC_real_ = 0.35, PCC_random_ = 0.20), growth cone (PCC_real_ = 0.31, PCC_random_ = 0.20)
**Figure S1D (N-terminal hnRNP R antibody from Sigma)**: Cell body (PCC_real_ = 0.66, PCC_random_ = 0.56), axon (PCC_real_ = 0.26, PCC_random_ = 0.19), growth cone (PCC_real_ = 0.26, PCC_random_ = 0.19)

For better visualization the ‘Colocalization Finder’ plugin of ImageJ was applied highlighting artificially superimposed colocalizing points calculated computationally.

### Immunohistochemial analysis of motor endplates

The *Diaphragm* muscle was dissected from E18, P4 or adult mice by carefully cutting alongside the ribs and thoroughly removing attached liver and lung tissue. The tissue was washed in PBS-T (0.1% Tween-20) for 20 min at RT. Blood clots and fasciae were carefully purged off the muscle tissue prior to fixation with 4% PFA at RT for 12 min (E18), 15 min (P4) or 20 min (adult stage), respectively. After incubation with ω-Bungarotoxin (Invitrogen, conjugated with Alexa488 or Alexa647, respectively) for 25 min at RT, the *Diaphragm* was incubated overnight at 4°C with a blocking solution comprising 2% BSA, 0.1% Tween-20 and 10% donkey serum or 15% goat serum, respectively. The tissue was then incubated with primary antibodies for three days at 4°C. After washing with PBS (pH 7.4, PAA Laboratories) thrice for 15 min each appropriate secondary antibodies were applied for 1 h at RT. Again, the tissue was washed three times with PBS for each 15 min, counterstained with DAPI and embedded in Aqua Polymount (Polysciences). For immunohistochemical analysis the following primary and secondary antibodies were used: monoclonal mouse anti-SMN (1∶250, BD Biosciences, 610646), polyclonal rabbit anti-hnRNP R (1∶2000, polypeptide antiserum aa1-18, ICN, Wuerzburg), polyclonal guinea pig anti-synaptophysin (1∶600, Synaptic Systems), goat anti-mouse (H+L) IgG_1_ (Cy5, 1∶500, Abcam ab136127), donkey anti-rabbit (H+L) IgG (Cy3, 1∶700, Jackson Immunoresearch 711-165-152), donkey anti-guinea pig (H+L) IgG (Cy2, 1∶400; Dianova 706-225-148 or Cy3, 1∶500; Dianova 706-166-148). Notably, a mouse monoclonal IgG_1_ antibody was used for immunodetection of Smn reducing unspecific signals derived from endogenous mouse antibodies and adhesion molecules which share great homology with immunoglobulins. For visualization of presynaptic hnRNP R or Smn, respectively, ‘planar’ endplates with prominent SynPhys staining and nuclei barely touching the BTX- and SynPhys-positive area were preferably imaged. For P4 and adult tissue the *Diaphragm* muscle was teased directly after fixation to improve antibody penetration.

### Immunohistochemical analysis of cross sections from native embryonic spinal cords

Spinal cords were isolated without vertebrae from E18 mouse embryos. Tissues were washed with PBS for 20 min at RT prior to fixation with 4% PFA for at least 2 h at RT. Spinal cords were kept in 30% sucrose solution overnight at 4°C. Spinal cords were embedded in Tissue Tek (O.C.T. Mount Medium, Sakura) and 10 µm thick cross cryosections were produced. Cross sections were washed with PBS and blocked with 10% donkey serum, 2% BSA and 0.3% TritonX for 1 h at RT. Then, primary antibodies against ChAT (anti-ChAT, 1∶100, Millipore, AB144P), Smn (anti-SMN, 1∶250, BD Biosciences, 610646) and hnRNP R (anti-hnRNP R polypeptide antiserum aa1-18, 1∶2000, ICN, Wuerzburg) were added overnight at 4°C. Cross sections were washed with PBS thrice and secondary antibodies (donkey anti-rabbit (H+L) IgG conjugated with Cy3, 1∶700, Jackson Immunoresearch 711-165-152; donkey anti-mouse (H+L) IgG conjugated with Alexa488, 1∶400, Invitrogen A-21202); donkey anti-goat (H+L) IgG conjugated with Cy5, 1∶300, Jackson Immunoresearch 705-175-003) were applied for 1 h at RT. After washing with PBS for three times cross sections were embedded in Aqua Polymount (Polysciences).

### Preparation and staining of cryostat sections of ventral roots and sciatic nerves

The *Gastrocnemius* was prepared as described previously [91]. Briefly, adult mice were perfused with 4% PFA and ventral roots were isolated, postfixed in 4% PFA overnight and transferred into buffer with increasing sucrose content, i.e. 10 to 30%. Afterwards, the tissue was embedded in Tissue Tek (O.C.T. Mount Medium, Sakura) and frozen within 2-methylbutane cooled by liquid N_2_. The ventral roots were cut in 10 µm thick cross cryosections. The sections were then stained as described above. The following primary and secondary antibodies were used: Smn (anti-SMN, 1∶250, BD Biosciences, 610646), hnRNP R (anti-hnRNP R polypeptide antiserum aa1-18, 1∶2000, ICN, Wuerzburg) and neurofilament (anti-neurofilament, 1∶500, AB5539, Millipore), goat anti-mouse (H+L) IgG conjugated with Cy3 (1∶200, Jackson Immunoresearch 115-165-003), swine anti-rabbit (H+L) IgG conjugated with FITC (1∶40, Dako, F0205) and goat anti-chicken (H+L) IgG conjugated with Cy5 (1∶400, ab6569, Abcam).

### Purification of murine recombinant hnRNP R and SMN protein

His-tagged hnRNP R and SMN full length proteins were expressed in *E. coli* after cloning the corresponding cDNA constructs into the pET-28a and pET-32a vector system (Novagen, Madison, WI), respectively. The expected molecular size of the His-Tag from this vector corresponds to 15 kDa. In line with this notion, the molecular sizes of the tagged proteins are 89.1 kDa for recombinant hnRNP R and 49.8 kDa for recombinant SMN. Both proteins were purified using 1 ml His-Trap HP and Superdex 10/300 gel filtration columns (GE Healthcare). The recombinant proteins were produced in the *E. coli* strain BL21 grown in MagicMedium (Invitrogen) for 6 hours at 30°C and for 18 hours at 18°C without further induction. Bacterial pellets were sonicated for 1–2 min in 50 mM sodium phosphate (pH 8.0), 500 mM NaCl, 20 mM imidazole, 5% (v/v) glycerol, 1 mM TCEP and protease inhibitor (Roche), and spinned for 30 min at 30 000 g. The clarified supernatants were loaded onto a 1 ml His-Trap HP column at 0.5 ml/min flow rate. The columns were washed for several hours with 50 mM sodium phosphate buffer (pH 8.0), 500 mM NaCl, 30 mM imidazole, 5% (v/v) glycerol and 0.5 mM TCEP at a flow rate of 1.0 ml/min. Bound proteins were eluted with 50 mM sodium phosphate buffer (pH 8.0), 500 mM NaCl, 250 mM imidazole, 5% (v/v) glycerol and 0.5 mM TCEP at a flow rate of 1.0 ml/min. In a final step eluted proteins were subjected to a size exclusion column using a Superdex 10/300 column that was run with 50 mM sodium phosphate buffer (pH 8.0), 50 mM NaCl and 5% (v/v) glycerol at a flow rate of 0.5 ml/min. Fractions and purified proteins were separated on 8% PAA gels and colloidial or silver stained. Whole purification was conducted on an Äckta FPLC system (GE Healthcare). To determine protein concentration spectrophotometric measurements were carried out with a Nanodrop (ND-1000, PeqLab). Image processing of colloidial stainings was carried out with Photoshop 7.0 (Adobe).

### Subcellular fractionation of mouse motoneurons

At least 100 000 primary motoneurons were plated on a 12-well cell culture dish and cultured for 7DIV in the presence of 10 ng/ml BDNF and CNTF. Buffers for fractionation were prepared freshly and filtered with a 0.45 µm filter. Cells were washed three times with ice-cold PBS. Motoneurons were lysed with the cytoplasmic fractionation buffer containing 50 mM Tris (pH 7.4), 150 mM NaCl, 0.1% NP-40, 1 mM MgCl_2_ and 1x Complete Protease inhibitor (Roche) for 10 min on ice. Cells were scrapped off thoroughly and centrifuged at 500 g for 10 min at 4°C. The supernatant, i.e. the cytoplasmic fraction, was collected. The pellet was washed three times with 25 µl cytoplasmic buffer to remove the remaining cytoplasmic fraction. Supernatants were collected and added to the existing cytoplasmic fraction.

The pellet was lysed with nuclear fractionation buffer comprising 20 mM HEPES (pH 7.4), 400 mM NaCl, 1 mM EDTA, 0.5 mM NaF, 0.5 mM DTT, 2.5% Glycerol, 0.6% CHAPS, 2 U/100 µl Benzonase and 1x Complete Protease Inhibitor (Roche) for 3 min on ice. The fraction was homogenized, incubated for 10 min on ice and centrifuged at 5000 g for 10 min at 4°C. The supernatant, i.e. the soluble nuclear fraction, was collected. Total protein concentration of nuclear and cytosolic fractions was assessed using the Pierce BCA Protein Assay Kit. Equal amounts of proteins were loaded for Western Blot analyses. Cytoplasmic and nuclear fractions were controlled using antibodies against GAPDH, α tubulin and histone H3 (for more information see the chapter *Western blotting*).

### Subcellular fractionation of E18 native spinal cord

Spinal cords without vertebrae from E18 mouse embryos were dissected and washed with PBS three times. Tissues were lysed with 200 µl cytoplasmic fractionation buffer (see above) for 5 min on ice. Spinal cords were homogenized and incubated for 5 min on ice prior to centrifugation at 500 g for 10 min at 4°C. Supernatants, i.e. cytoplasmic fraction, were collected. In turn, the pellets were lysed with 100 µl nuclear fractionation buffer (see above) for 3 min on ice. Again, the pellets were homogenized and incubated for 10 min on ice. The lysed fractions were centrifuged at 10 000 g for 10 min at 4°C. The supernatants were collected serving as soluble nuclear fractions. The insoluble nuclear fraction was redissolved with RIPA Buffer and further analyzed. Total protein concentration of nuclear and cytosolic fractions was assessed using the Pierce BCA Protein Assay Kit. For Western Blot analyses equal amounts of protein were loaded onto the gel. The purity of the obtained fractions was controlled by GADPH, α tubulin and histone H3 (for more information see the chapter *Western blotting*).

### Coimmunoprecipitation of recombinant proteins

The association between recombinant hnRNP R and SMN was analyzed by coimmunoprecipitation using GammaBind Plus Sepharose beads (GE Healthcare). 250 or 500 ng of rhnRNP R and 250 ng of rSMN were incubated in binding buffer, comprising 50 mM sodium phosphate (pH 8.0), 5% (v/v) glycerol, 50 mM NaCl and 0.1% Tween, with 20 µl Sepharose beads and 1 µg antibodies against hnRNP R (ab30930, Abcam), SMN (610647, BD Bioscience) or non-specific IgG control (anti-GFP, sc-8334, Santa Cruz) for 1 h at RT. The resin was washed 5 times with binding buffer to remove unbound proteins. For elution beads were boiled in 2xLaemmli buffer at 95°C for 5 min. The eluted proteins were then analyzed by Western blotting (for more information see the chapter *Western blotting*). Notably, Light chain-specific secondary antibodies (Jackson Immunoresearch) were used for detection since the 55 kDa heavy chain from the immunoprecipitation would mask the SMN signal.

### Immunoprecipitation

Spinal cord without vertebra isolated from E18 mouse embryo or approximately 500 000 primary motoneurons cultured for 7DIV were used for coimmunoprecipitation experiments. Nuclear and cytoplasmic proteins were extracted (see above). Fractions were pre-cleaned with protein G beads (for rabbit IgG antibody) and protein A beads (for mouse IgG antibody) for 1 h. Afterwards, the pre-cleaned lysate was incubated with 5 µl rabbit anti-hnRNP R (abcam, ab30930), 4 µl anti-Smn (BD Biosciences, 610646) and consistent rabbit and mouse FLAG antibodies, respectively as negative control for 6 h under rotary agitation at 4°C. Protein G-agarose beads (Roche) for rabbit antibody and protein A-agarose beads (Roche) for mouse were washed with PBS and equilibrated with lysis buffer. The protein and antibody lysate were added to the respective equilibrated beads and incubated for 1 h under rotary agitation at 4°C. Subsequently, samples were centrifuged at 500 g for 5 min and the supernatant was removed. Then, beads were washed thrice with the appropriate lyses buffer and finally with PBS. The proteins were eluted by boiling the beads with 2x Laemmli buffer at 90°C for 10 min. Immunoblotting was performed for hnRNP R and Smn to confirm coimmunoprecipitation.

### Western blotting

Primary motoneurons or E18 spinal cord tissue, respectively, were lysed with cytosolic and nuclear fractionation buffer, solubilized in Laemmli buffer (125 mM Tris, pH 6.8, 4% SDS, 10% β-mercaptoethanol, 20% glycerol, and 0.004% bromophenol blue) and boiled for 10 min at 99°C. Proteins were then subjected to SDS-PAGE, blotted onto PVDF membrane, incubated with the corresponding antibodies, and developed with either ECL or ECL Advance Systems (GE Healthcare) on X-ray film (Fuji super RX). Western blots were scanned and quantified by densitometry analysis with ImageJ (National Institutes of Health). For Western Blot analysis the following primary and secondary antibodies were used: anti-SMN (BD Biosciences, 610646, 1∶3000), anti-hnRNP R (Abcam, ab30930, 1∶3000 or polypeptide antiserum aa1-18, ICN, Wuerzburg, 1∶3000), anti-GFP (Santa Cruz, sc-8334, 1∶4000), anti-GAPDH (Millipore, 6C5, 1∶4000), anti-α tubulin (T5168, Sigma, 1∶4000), anti-histone H3 (Abcam, ab8580, 1∶20 000), anti-calnexin (Abcam, ab22595, 1∶5000), anti-GFP (Santa Cruz, sc-8334, 1∶4000), anti-mouse IgG (Jackson Immunoresearch, 115-035-003, 1∶10000), anti-rabbit IgG (Jackson Immunoresearch, 111-035-003, 1∶10000), anti-mouse light chain-specific (Jackson Immunoresearch, 111-035-174, 1∶10000) and anti-rabbit light chain-specific (Jackson Immunoresearch, 211-032-171, 1∶10000).

### Supplementary Material

Supplementary Material is available online at the *PLOS ONE* homepage ‘www.plosone.org’.

## Supporting Information

Figure S1 **Structure of hnRNP R protein and validation of N-terminal hnRNP R antibody.** (A) HnRNP R contains three RNA-recognition motifs (RRM) and an arginine- and glycine-rich domain. ICN 1-18 binds to the very N-terminal region of hnRNP R in contrast to other antibodies which bind to the C-terminus. (B) Two shRNA binding sites were designed to deplete hnRNP R protein. Thereby, the one near the 3′UTR was used in this study since it affects all predicted hnRNP R isoforms identified by database research [92]. The other lentiviral construct was applied and verified as previously reported [29]. (C) Representative images of GFP- and sh-hnRNP R-infected motoneurons cultured for 7DIV on laminin-111 and stained against hnRNP R, Smn and DAPI (scale bar: 10 µm). Using an independent N-terminal hnRNP R antibody a significant reduction (P = 0.0272, t = 5.941, DF = 2) of hnRNP R immunoreactivity of 52% was detected in sh-hnRNP R-infected motoneuron cell bodies (0.48±0.09, n = 3, N = 40) in comparison to GFP-infected control cells (set as ‘1’, n = 3, N = 57). Notably, loss of hnRNP R did not significantly alter cytosolic Smn signal intensity (sh-hnRNP R 0.82±0.08, P = 0.1426, t = 2.356, DF = 2) and the number of Smn-positive Gems (GFP 0.86±0.24; sh-hnRNP R 1.03±0.24; P = 0.1182, t = 2.645, DF = 2). (D) Pattern and subcellular distribution of hnRNP R in cell bodies, axons and axonal growth cones, using the independent N-terminal hnRNP R antibody, were similar to the results obtained with the ICN 1-18 with a relatively stronger staining in the nucleus. Motoneurons were cultured for 5DIV on laminin-111. Colocalization analysis of Smn and hnRNP R revealed also comparable results in soma (PCC 0.66±0.02, MOC 0.70±0.01, N = 6), axon (PCC 0.26±0.02, MOC 0.48±0.01, N = 7) and axonal growth cone (PCC 0.26±0.05, MOC 0.47±0.03, N = 7), as highlighted in white (right panel) (scale bar: soma, 10 µm; axon and growth cone, 5 µm).
(TIF)

Figure S2 **Localization of Smn and hnRNP R in axon terminals and motor axons** ***in vivo.*** (A, B) Single optical slices with 1 µm step size and the corresponding maximum projections from P4 *Diaphragm* whole mount preparations stained against ω-BTX, DAPI and (A) Smn or (B) hnRNP R, respectively (scale bar: 5 µm). Both (A) Smn and (B) hnRNP R immunoreactivity coresided and co-occurred with presynaptic marker SynPhys. (C) Cross sections from adult sciatic nerve immunostained against hnRNP R, Smn and neurofilament (NF) (scale bar: 5 µm). Superimposed colocalizing points are highlighted in white.
(TIF)

Figure S3 **Loss of hnRNP R immunoreactivity after preabsorption with recombinant protein.** (A) hnRNP R signal was highly reduced after preabsorption of ICN 1-18 with recombinant hnRNP R protein (B), whereas pre- and postsynaptic structures were visible, as indicated by synaptophysin and BTX staining, respectively. DAPI staining showed synaptic nuclei or nuclei from non-neuronal cells, respectively (scale bar: 5 µm).
(TIF)
