# Supplementary figures and images for: Presynaptic Localization of Smn and hnRNP R in Axon Terminals of Embryonic and Postnatal Mouse Motoneurons
Source: PLoS One. 2014 Oct 22;9(10):e110846. doi: 10.1371/journal.pone.0110846 (PMC4206449; doi:10.1371/journal.pone.0110846)

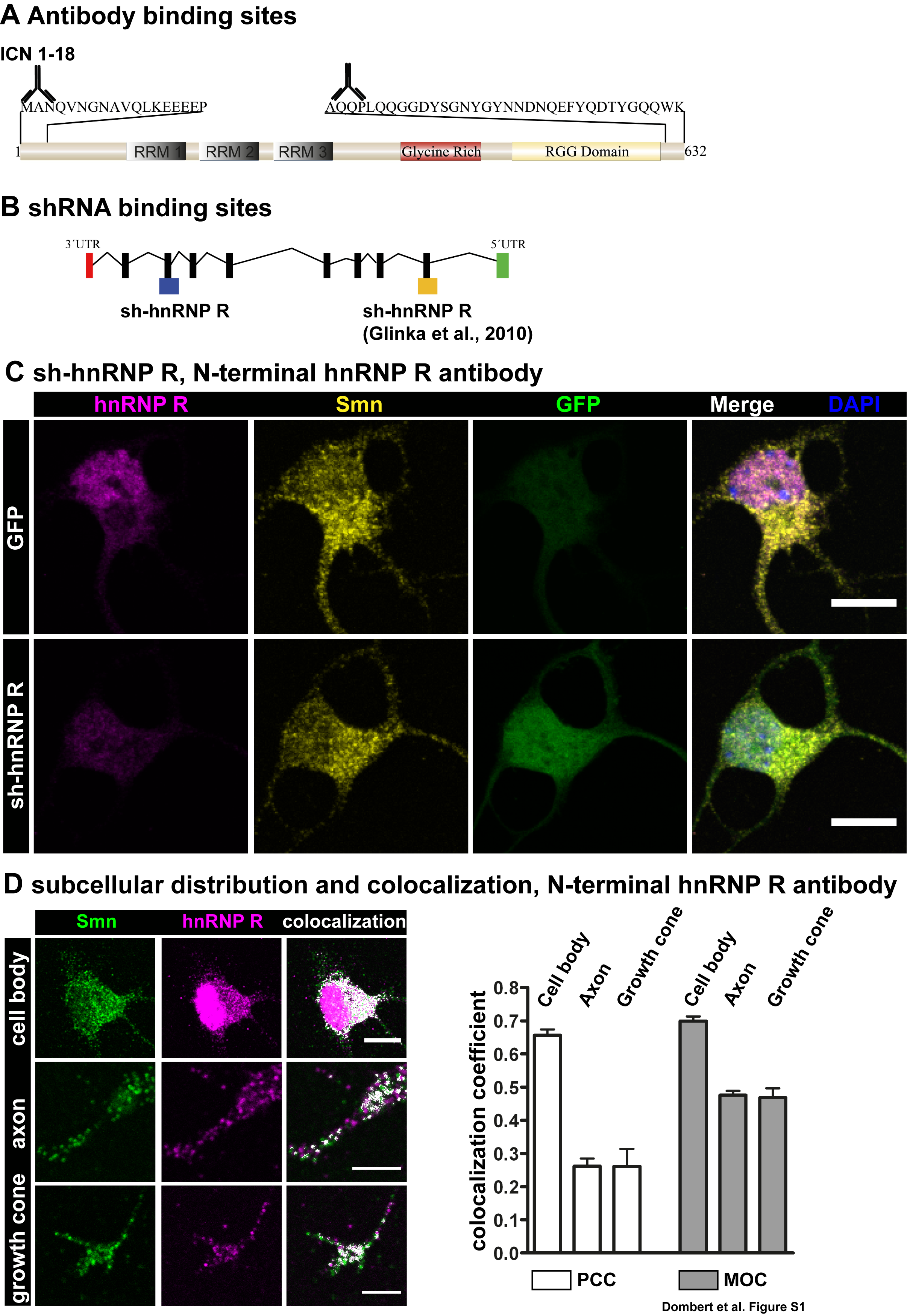

Supplement: Figure S1 — Structure of hnRNP R protein and validation of N-terminal hnRNP R antibody. (A) HnRNP R contains three RNA-recognition motifs (RRM) and an arginine- and glycine-rich domain. ICN 1-18 binds to the very N-terminal region of hnRNP R in contrast to other antibodies which bind to the C-terminus. (B) Two shRNA binding sites were designed to deplete hnRNP R protein. Thereby, the one near the 3′UTR was used in this study since it affects all predicted hnRNP R isoforms identified by database research [92]. The other lentiviral construct was applied and verified as previously reported [29]. (C) Representative images of GFP- and sh-hnRNP R-infected motoneurons cultured for 7DIV on laminin-111 and stained against hnRNP R, Smn and DAPI (scale bar: 10 µm). Using an independent N-terminal hnRNP R antibody a significant reduction (P = 0.0272, t = 5.941, DF = 2) of hnRNP R immunoreactivity of 52% was detected in sh-hnRNP R-infected motoneuron cell bodies (0.48±0.09, n = 3, N = 40) in comparison to GFP-infected control cells (set as ‘1’, n = 3, N = 57). Notably, loss of hnRNP R did not significantly alter cytosolic Smn signal intensity (sh-hnRNP R 0.82±0.08, P = 0.1426, t = 2.356, DF = 2) and the number of Smn-positive Gems (GFP 0.86±0.24; sh-hnRNP R 1.03±0.24; P = 0.1182, t = 2.645, DF = 2). (D) Pattern and subcellular distribution of hnRNP R in cell bodies, axons and axonal growth cones, using the independent N-terminal hnRNP R antibody, were similar to the results obtained with the ICN 1-18 with a relatively stronger staining in the nucleus. Motoneurons were cultured for 5DIV on laminin-111. Colocalization analysis of Smn and hnRNP R revealed also comparable results in soma (PCC 0.66±0.02, MOC 0.70±0.01, N = 6), axon (PCC 0.26±0.02, MOC 0.48±0.01, N = 7) and axonal growth cone (PCC 0.26±0.05, MOC 0.47±0.03, N = 7), as highlighted in white (right panel) (scale bar: soma, 10 µm; axon and growth cone, 5 µm). (TIF) [file pone.0110846.s001.tif]

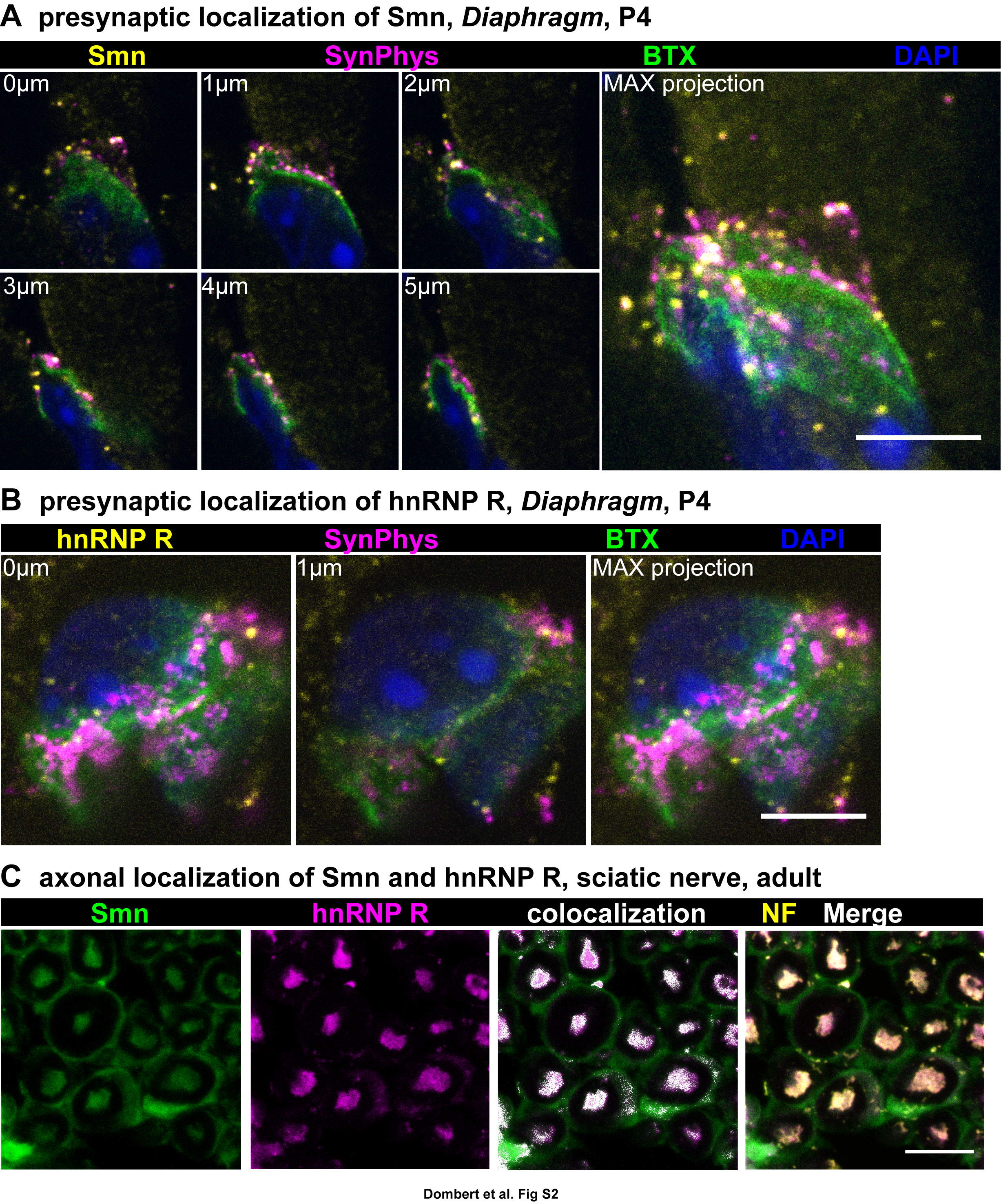

Supplement: Figure S2 — Localization of Smn and hnRNP R in axon terminals and motor axons in vivo. (A, B) Single optical slices with 1 µm step size and the corresponding maximum projections from P4 Diaphragm whole mount preparations stained against ω-BTX, DAPI and (A) Smn or (B) hnRNP R, respectively (scale bar: 5 µm). Both (A) Smn and (B) hnRNP R immunoreactivity coresided and co-occurred with presynaptic marker SynPhys. (C) Cross sections from adult sciatic nerve immunostained against hnRNP R, Smn and neurofilament (NF) (scale bar: 5 µm). Superimposed colocalizing points are highlighted in white. (TIF) [file pone.0110846.s002.tif]

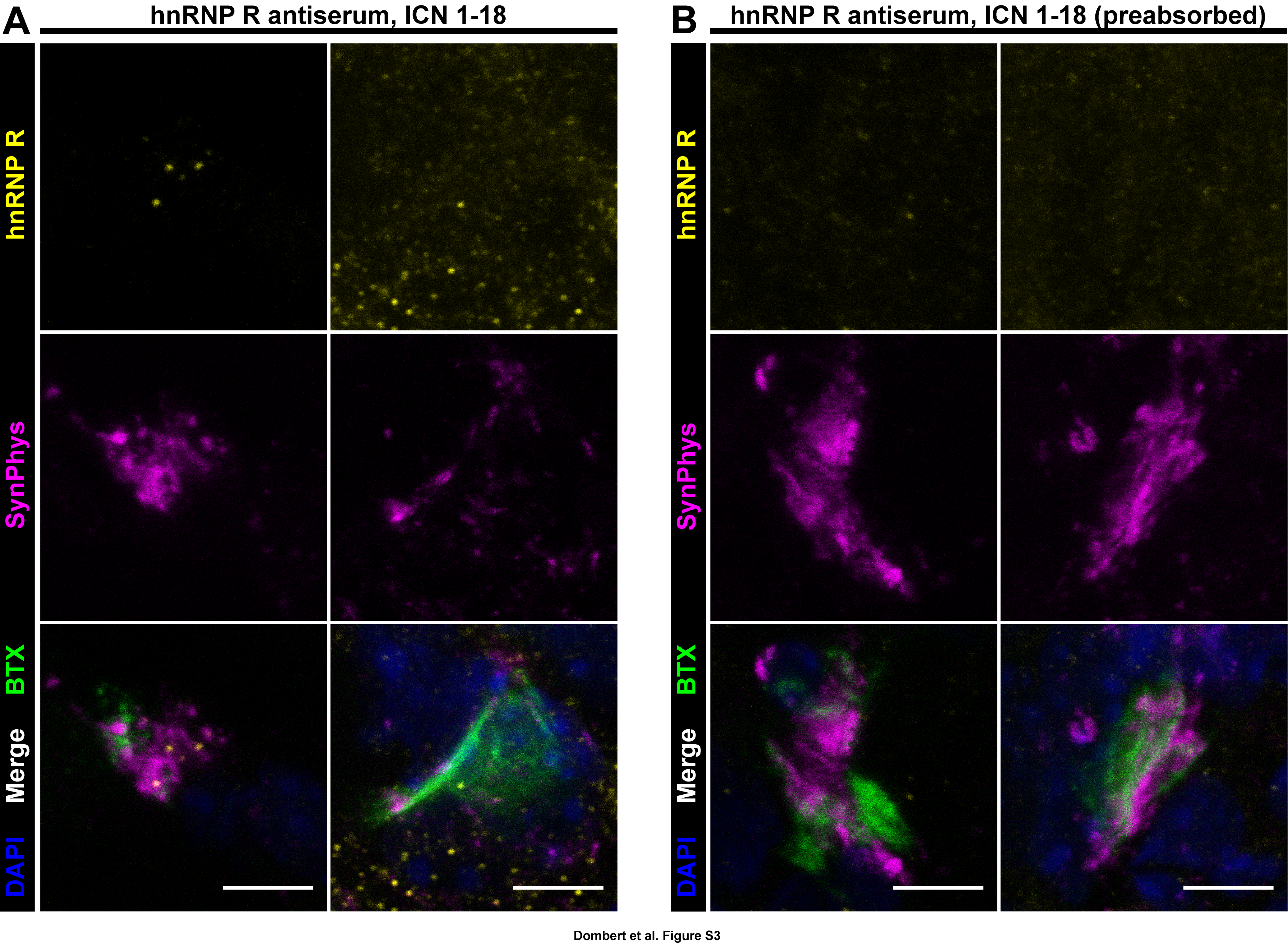

Supplement: Figure S3 — Loss of hnRNP R immunoreactivity after preabsorption with recombinant protein. (A) hnRNP R signal was highly reduced after preabsorption of ICN 1-18 with recombinant hnRNP R protein (B), whereas pre- and postsynaptic structures were visible, as indicated by synaptophysin and BTX staining, respectively. DAPI staining showed synaptic nuclei or nuclei from non-neuronal cells, respectively (scale bar: 5 µm). (TIF) [file pone.0110846.s003.tif]
